# Identification of shared pathogenetic mechanisms between COVID-19 and IC through bioinformatics and system biology

**DOI:** 10.1038/s41598-024-52625-z

**Published:** 2024-01-24

**Authors:** Zhenpeng Sun, Li Zhang, Ruihong Wang, Zheng Wang, Xin Liang, Jiangang Gao

**Affiliations:** 1 Department of Urology, Qingdao Municipal Hospital, No.5, Donghai Middle Road, Shinan District, Qingdao, 266001 Shandong China; 2 Institute of Systems Medicine, Chinese Academy of Medical Sciences, Peking Union Medical College, Beijing, China; 3 Suzhou Institute of Systems Medicine, Suzhou, China; 4 Department of Outpatient, Qingdao Central Hospital, Qingdao University, Qingdao, China; 5 Zhucheng People’s Hospital, Zhucheng, China; 6 Qingdao Medical College, Qingdao University, Qingdao, China

**Keywords:** Viral infection, Diseases, Immunological disorders, Infectious diseases, Respiratory tract diseases, Urogenital diseases, Computational biology and bioinformatics, Data mining, Data processing, Gene regulatory networks, Virtual drug screening, Viral infection, Autoimmune diseases, Inflammatory diseases, Immunology, Chemokines, Cytokines, Immunological disorders, Infectious diseases, Inflammation, Bladder, Bladder disease

## Abstract

COVID-19 increased global mortality in 2019. Cystitis became a contributing factor in SARS-CoV-2 and COVID-19 complications. The complex molecular links between cystitis and COVID-19 are unclear. This study investigates COVID-19-associated cystitis (CAC) molecular mechanisms and drug candidates using bioinformatics and systems biology. Obtain the gene expression profiles of IC (GSE11783) and COVID-19 (GSE147507) from the Gene Expression Omnibus (GEO) database. Identified the common differentially expressed genes (DEGs) in both IC and COVID-19, and extracted a number of key genes from this group. Subsequently, conduct Gene Ontology (GO) functional enrichment and Kyoto Encyclopedia of Genes and Genomes (KEGG) enrichment analysis on the DEGs. Additionally, design a protein–protein interaction (PPI) network, a transcription factor gene regulatory network, a TF miRNA regulatory network, and a gene disease association network using the DEGs. Identify and extract hub genes from the PPI network. Then construct Nomogram diagnostic prediction models based on the hub genes. The DSigDB database was used to forecast many potential molecular medicines that are associated with common DEGs. Assess the precision of hub genes and Nomogram models in diagnosing IC and COVID-19 by employing Receiver Operating Characteristic (ROC) curves. The IC dataset (GSE57560) and the COVID-19 dataset (GSE171110) were selected to validate the models' diagnostic accuracy. A grand total of 198 DEGs that overlapped were found and chosen for further research. FCER1G, ITGAM, LCP2, LILRB2, MNDA, SPI1, and TYROBP were screened as the hub genes. The Nomogram model, built using the seven hub genes, demonstrates significant utility as a diagnostic prediction model for both IC and COVID-19. Multiple potential molecular medicines associated with common DEGs have been discovered. These pathways, hub genes, and models may provide new perspectives for future research into mechanisms and guide personalised and effective therapeutics for IC patients infected with COVID-19.

## Introduction

Interstitial cystitis (IC) is a chronic nonbacterial inflammatory bladder disease of unknown etiology, affecting millions of American women with an incidence rate of approximately 2%^1^. Its primary clinical manifestations encompass symptoms of lower urinary tract hypersensitivity, including bladder pain or discomfort, urgency, and frequency of urination^2^. The exact mechanisms of IC are not well comprehended, although there are several components thought to have major involvement, including changes in epithelial permeability, mast cell activation, upregulation of sensory afferent nerves, inflammation, and autoimmunity^3,4^.

Coronavirus disease 2019 (COVID-19), caused by severe acute respiratory syndrome coronavirus 2 (SARS-CoV-2)^5^, is an infectious disease that presents with symptoms such as fever, cough, muscle pain, and weariness. It can also cause serious damage to many organs^6,7^. According to data from the World Health Organization (WHO) (https://covid19.who.int/), as of July 20, 2023, COVID-19 had caused over 760 million infections and more than 6 million fatalities. It has been observed that SARS-CoV can be transmitted through urine^8^. In the acute phase of a COVID-19 infection, individuals may experience sudden urinary frequency and urgency, persisting for several weeks^9,10^. Lamb et al.^11^ found that patients with confirmed SARS-CoV-2 infection are accompanied by pronounced and enduring genitourinary symptoms. Furthermore, they observed elevated levels of proinflammatory cytokines in the patients' urine samples. These associated urinary symptoms are called COVID-19-Associated Cystitis (CAC).

Angiotensin-Converting Enzyme 2 (ACE2) is the primary receptor identified by SARS CoV-2. It is highly expressed in the bladder, making the bladder susceptible to assault during COVID-19 infection^12^. Previous reports have found that COVID-19 is linked to immune disorders and abnormal inflammatory responses^13,14^. According to recent studies, SARS infection can activate neutrophils and exacerbate local inflammatory damage through the release of neutrophil extracellular traps (NETs)^15,16^. However, the relationship between COVID-19 and cystitis remains unclear. It is necessary to further explore their potential associations and molecular mechanisms to develop effective treatment approaches for cystitis caused by or aggravated by SARS-CoV-2 infection.

**Figure 1:**
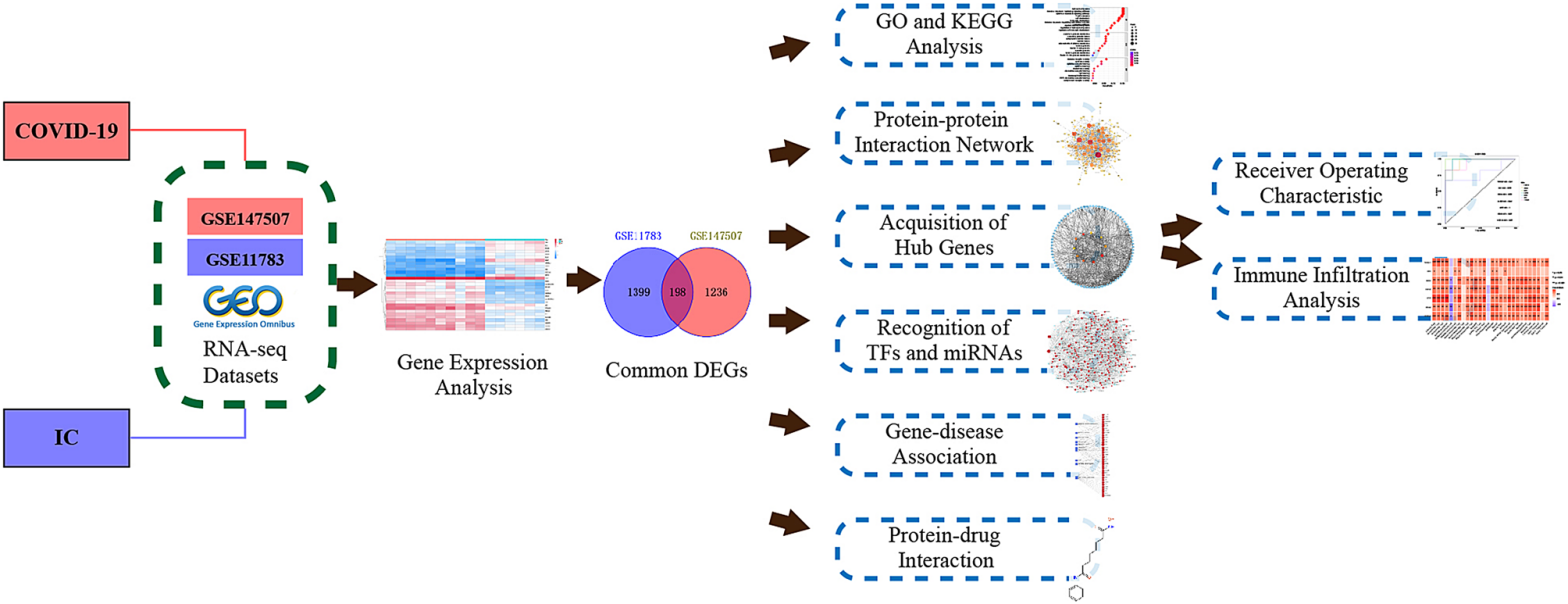
An illustration in schematic form of the study's entire procedure.

This study aimed to explore the pathogenesis and genetic correlation between cystitis and COVID-19. Herein, we selected the GSE11783 and GSE147507 datasets from the NCBI-Gene Expression Omnibus database (NCBI-GEO). Bioinformatics and machine learning algorithms were used to identify common differentially expressed genes (DEGs) and determine the hub genes in the IC patients and COVID-19 patients. Protein–protein interaction networks (PPI), transcription factor (TF) gene regulatory networks, and microRNA (miRNA) gene regulatory networks were constructed based on the shared DEGs, which contribute to the development of IC and COVID-19. Furthermore, we screened potential drugs targeting the DEGs. Finally, we analyzed the relationship between the hub genes and immune infiltration. The central genes may offer novel insights for future research on the biological mechanisms underlying both IC and COVID-19, and potentially lead to the discovery of new therapeutic targets. Figure 1 provides a detailed depiction of the study's specific methodology.

## Materials

### Acquisition of the datasets

We accessed the GEO database (https://www.ncbi.nlm.nih.gov/geo/; accessed on July 17, 2023) to procure RNA-sequencing datasets pertaining to IC and COVID-19^17^. The GSE147507 dataset comprises a transcriptional study of 78 homo sapiens samples, consisting of 23 COVID-19 cases and 55 healthy control samples. The RNA sequence from these samples was obtained using the GPL18573 Illumina NextSeq 500. The other dataset, GSE11783, utilizes the GPL570 [HG-U133_Plus_2] Affymetrix Human Genome U133 Plus 2.0 Array to conduct a transcriptional analysis. It includes a total of 16 samples, with 10 cases of IC and 6 healthy control samples. The above two datasets were selected as training sets. In addition, the GSE171110 and GSE57560 datasets were selected as the test sets for this study. Given that our study focuses exclusively on Homo sapiens, we excluded the high-throughput sequencing data of ferrets from the COVID-19 dataset, GSE147507, within this investigation.

### Identification of DEGs and common DEGs between COVID-19 and IC

To unveil DEGs in the GSE147507 and GSE11783 datasets, we scrutinized COVID-19 vs. non-COVID-19 and IC vs. normal states using the "limma" package (version 4.1.1) by R^18^. The Benjamini–Hochberg False Discovery Rate (FDR) approach was employed to narrow down genes and maintain statistical significance. The criterion used was Padj < 0.05 and |log2FoldChange (FC)| > 1. The heatmaps and volcano plots were constructed using the "Pheatmap", "Enhancedvolcano”, and “ggplot2” packages. The common DEGs across the two datasets were identified using an online analysis tool called Venny2.1 (https://bioinfogp.cnb.csic.es/tools/venny/).

### Functional enrichment analysis of DEGs

In order to determine the biological function categories and mechanisms of the common DEGs, we employed R's "clusterprofiler"^19^ to evaluate the Kyoto Encyclopedia of Genes and Genomes (KEGG)^20–22^ analysis and Gene Ontology (GO)^23^ analysis, which encompass biological processes, cellular components, and molecular functions.

### Protein–protein interaction network analysis

PPIs are fundamental to several biological processes, providing insights into the physical and functional connections between proteins. We utilized STRING (version 12.0) (https://cn.string-db.org/) to generate a PPI network with a confidence threshold of 0.4 based on the DEGs that were uploaded^24^. The above results were imported into Cytoscape (version 3.9)^25^ software for visualization, where the color and size of nodes represent scores obtained from degree topology analysis. We employed CytoHubba, a plugin for Cytoscape (https://apps.cytoscape.org/apps/cytohubba), to detect hub genes within the PPI networks^26^. CytoHubba offers eleven distinct methods for detecting core components by analyzing various network characteristics. We utilized four different approaches, including Maximal Clique Centrality (MCC), Degree, Maximumc Neighborhood Component (MCN), and Closeness, to identify the top ten genes using each strategy. Finally, we utilized the overlapping portion of these four gene sets to acquire the most beneficial hub genes.

### Construction of TF‑gene and miRNA‑gene regulatory networks

To unravel the intricate transcriptional landscape and pivotal regulators governing common DEGs, we conducted a comprehensive investigation by integrating DEG-miRNA and DEG-TF interactions. Statistical analyses, visualizations, and meta-analyses of web-based gene expression data were facilitated using the widely embraced online platform, NetworkAnalyst (https://www.networkanalyst.ca/)^27^. The analysis was centered on NetworkAnalyst, which enabled the identification of structurally reliable TFs from the JASPAR database, which is renowned for its comprehensive multi-species TF binding profiles^28^. MiRTarBase and TarBase offer data on miRNA-target interactions that have undergone experimental validation^29,30^. We accessed the aforementioned databases through the NetworkAnalyst platform and respectively constructed the networks between the DEGs and miRNAs. Subsequently, we determined the areas where the two networks intersected in order to underline their structural importance and especially highlight the miRNAs associated with the shared DEGs. The above results were visualized using Cytoscape (version 3.9) software.

### Gene-disease association analysis

DisGeNET is an extensive database that consolidates gene information linked to diseases from several literature sources^31^. Use the NetworkAnalyst platform to access the DisGeNET database and establish the relationship between genes and diseases. Gaining a comprehensive grasp of the molecular intricacies of the associated diseases can aid in identifying comorbidities and advancing our comprehension of these diseases.

### Evaluation of applicant drugs

The Drug Signatures Database (DSigDB) was utilized to identify small compounds that can downregulate hub genes, which has a comprehensive collection of 22,527 gene sets^32^. The Enrichr platform (https://amp.pharm.mssm.edu/Enrichr/) provided smooth access to the DSigDB repository^33^. We discovered drug entities inside the DSigDB database using the Enrichr framework, based on the detected DEGs. Through a methodical approach, prospective pharmacological molecules were identified that had the ability to influence the expression of key genes. This discovery might possibly provide focused therapeutic interventions.

### Immune cell infiltration and its correlation with hub genes

We systematically collected immune checkpoint genes (ICGs) from existing literature sources^34^. Single sample gene set enrichment analysis (ssGSEA) was performed on 28 different immune cell types using the R package "GSVA"^35^, and obtained the immunological enrichment scores for various immune cells. We employed the "ggboxplot" software package to graphically depict the complex correlation between the expression of immune checkpoints and the enrichment scores of these 28 immune cells in COVID-19 and IC. In addition, we employed the "ggcorrplot" package to calculate the correlation between the expression of the seven Hub genes and the infiltration of immune cells and subsequently visualized this link using the "ggplot2" package.

### ROC curve and correlation analysis of hub genes

Receiver operating characteristic (ROC) curve analysis was conducted to evaluate the predictive efficacy of individual hub genes. Utilizing the R-based pROC package^36^, we calculated the area under the ROC curve (AUC) and graphically represented these curves. Hub genes are deemed to have a general diagnostic predictive value when AUC > 0.5 and a superior diagnostic predictive value when AUC > 0.7. We also harnessed the "Corrplot" software package for correlation matrix visualization to discern interrelationships, enabling comprehensive scrutiny of hub gene correlations.

### Statistical analysis

Conduct statistical analysis with R (version 4.3.1). Continuous variables are employed for comparison across different groups, and t-tests are utilized for comparing variables that adhere to a normal distribution. Investigate the relationship between infiltrating immune cells and gene biomarkers using Spearman correlation analysis. A P-value < 0.05 indicates statistical significance.

## Results

### Identification of the common DEGs of COVID-19 and IC

**Figure 2:**
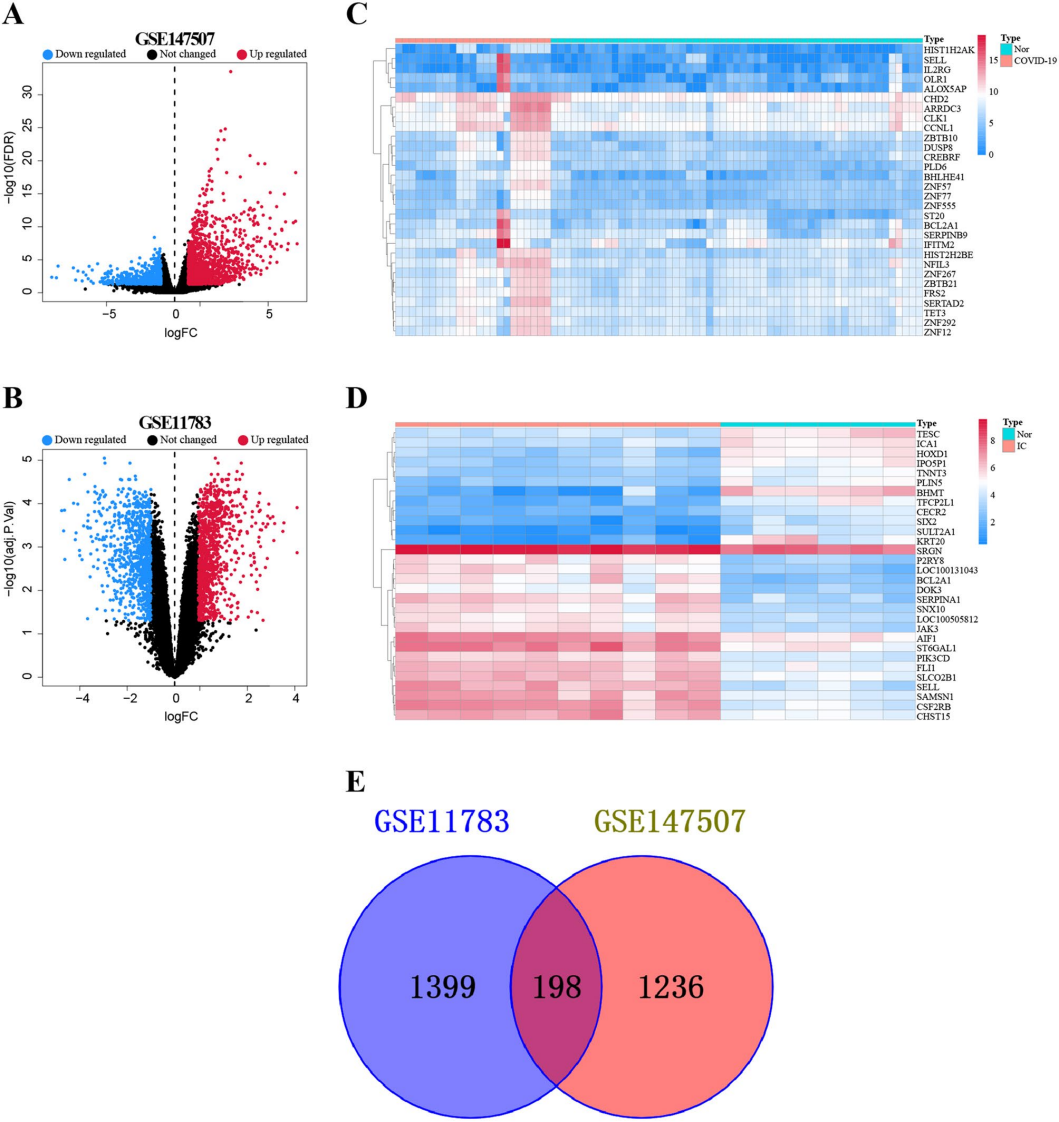
Differentially expressed genes (DEGs) of (**A**) COVID-19 and (**B**) IC are shown on volcano plots. With |log2(FC)| > 1 and a P-value < 0.05, red dots denoted up-regulated genes, blue dots denoted down-regulated genes, and grey dots denoted non-DEGs. The results of clustering analysis based on DEGs for (**C**) COVID-19 and (**D**) IC are displayed in heatmaps. A venn diagram then displayed the areas of GSE147507 and GSE11783 that overlapped.

The gene expression level of the selected datasets with adjusted batch effect is standardized, and the results before and after standardization are shown in Supplementary Fig. S1. Based on the criteria (Padj < 0.05 and |log2FoldChange (FC)| > 1), GSE147507 identified a total of 2016 DEGs (1431 upregulated and 585 downregulated genes), and GSE11783 identified 1926 DEGs (1045 upregulated and 881 downregulated genes). The DEGs between COVID-19 patients and healthy samples were shown using a heatmap and a volcano plot (Fig. 2A,B). The distribution of DEGs between patients with IC and controls was presented in Fig. 2C,D. A Venn diagram rendered the intersection of DEGs, yielding 198 common DEGs (Fig. 2E). The results of the differential expression study indicated potential shared mechanisms or interactions between COVID-19 and IC.

### Pathway enrichment and gene ontology analysis

**Figure 3:**
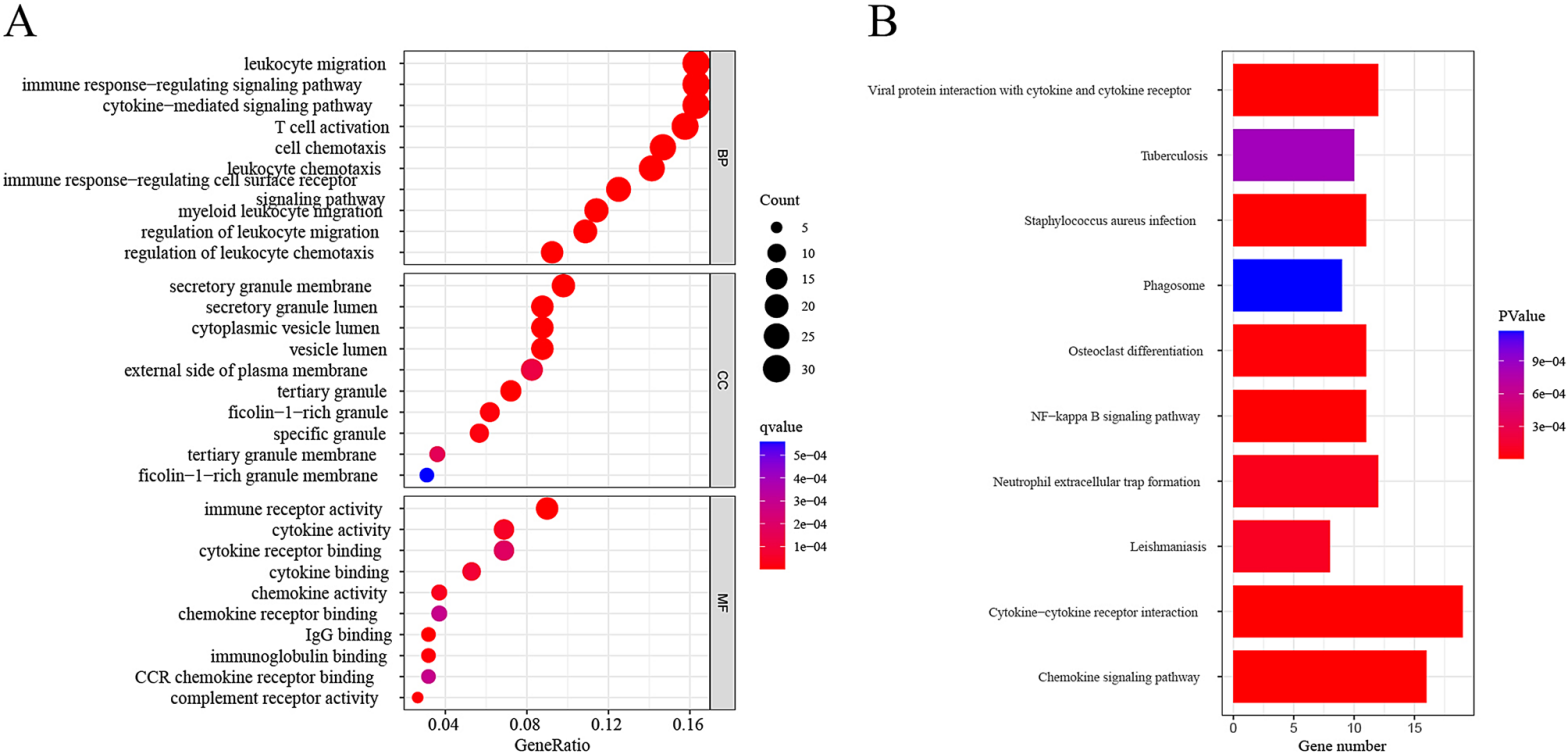
Functional analysis of IC and COVID-19. (**A**) The histogram of the GO enrichment analysis; the letters BP, CC, and MF stand for biological process, cellular component, and molecular function, respectively. (**B**) KEGG pathway analysis bar plot.

GO analysis and KEGG analysis provide insights into the biological traits and enrichment pathways of the shared DEGs. The bubble chart displays the top ten elements of GO terminology for each category. The DEGs showed considerable enrichment in immune response-regulating signaling pathways and cytokine-mediated signaling pathways within the biological processes (BP) subgroup. Within the cell composition (CC) subgroup, these DEGs were implicated in the secretory granule membrane and cytoplasmic vesicle lumen. In addition, in the molecular function (MF) subgroup, the DEGs were shown to be linked to immune receptor activity and cytokine activity, underscoring their pivotal roles in the immune system (Fig. 3A). The KEGG entichment analysis revealed that the common DEGs showed significant associations with the chemokine signaling pathway, cytokine–cytokine receptor interaction, neutrophil extracellular trap formation, NF-kappa B signaling pathway, viral protein interaction with cytokine, and cytokine receptor (Fig. 3B). The findings indicate that IC and COVID-19 patients exhibit an enrichment of inflammation and immune-related pathways.

### Construction of PPI and acquisition of hub genes

**Figure 4:**
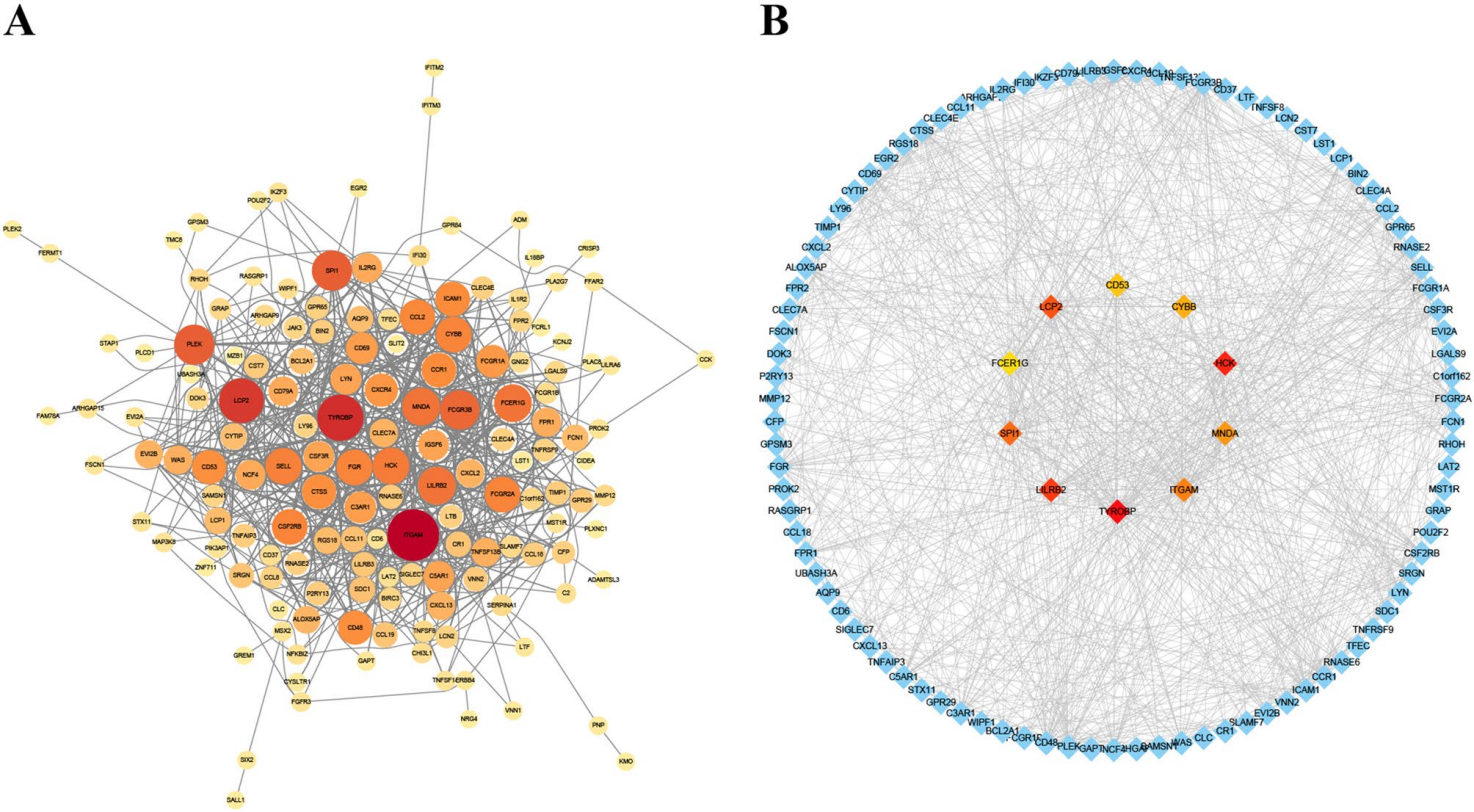
Common genes in COVID-19 and IC are analysed using the PPI network and clustering methods. Based on the STRING web database, (**A**) a network visualisation of 198 common genes using Cytoscape. (**B**) The Cytoscape MCC algorithm located the crucial cluster.

**Figure 5:**
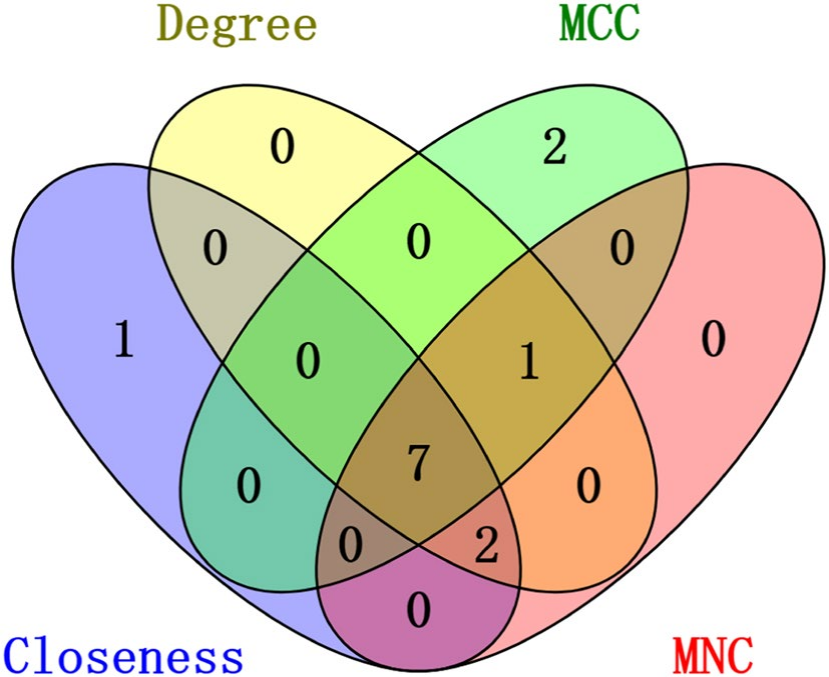
The Venn diagram displayed 7 hub genes that were tested by 4 different methods.

A PPI network has potential for identifying diseases genes, predicting gene function, and providing therapeutic insights. We employed the online analysis tool STRING to create a PPI network based on the shared DEGs to elucidate the connections between COVID-19 and IC, and the visualization was achieved through Cytoscape (v. 3.9). Figure 4A illustrates the PPI network, which consists of 153 nodes and 1024 edges. Circles' size and colour indicate the degree of protein interaction, with larger size and more intense colour representing higher centrality and greater relevance. Key genes were sieved via the CytoHubba package within Cytoscape. MCC method has superior precision in identifying critical proteins from the PPI network^26^. It identifies the ten most influential genes (Fig. 4B). In addition, we also utilized Degree, MCN, and Closeness algorithms to identify the top 10 hub genes, respectively (Table 1). Seven common key genes were found in these four hub gene sets, including FCER1G, ITGAM, LCP2, LILRB2, MNDA, SPI1, and TYROBP, which were considered core targets of IC and COVID-19 (Fig. 5). Further information on the obtained PPI network can be found in Supplementary Table S1.

**Table 1:** The leading ten hub genes identified by cytoHubba.

Closeness	Degree	MCC	MNC
FCER1G	PLEK	ITGAM	PLEK
FCGR3B	HCK	TYROBP	HCK
ITGAM	LCP2	LCP2	LCP2
LCP2	FCER1G	SPI1	FCER1G
LILRB2	TYROBP	MNDA	TYROBP
MNDA	SPI1	LILRB2	SPI1
PLEK	ITGAM	FCER1G	ITGAM
SELL	LILRB2	HCK	LILRB2
SPI1	MNDA	CYBB	MNDA
TYROBP	FCGR3B	CD53	FCGR3B

### Construction of the regulatory network

**Figure 6:**
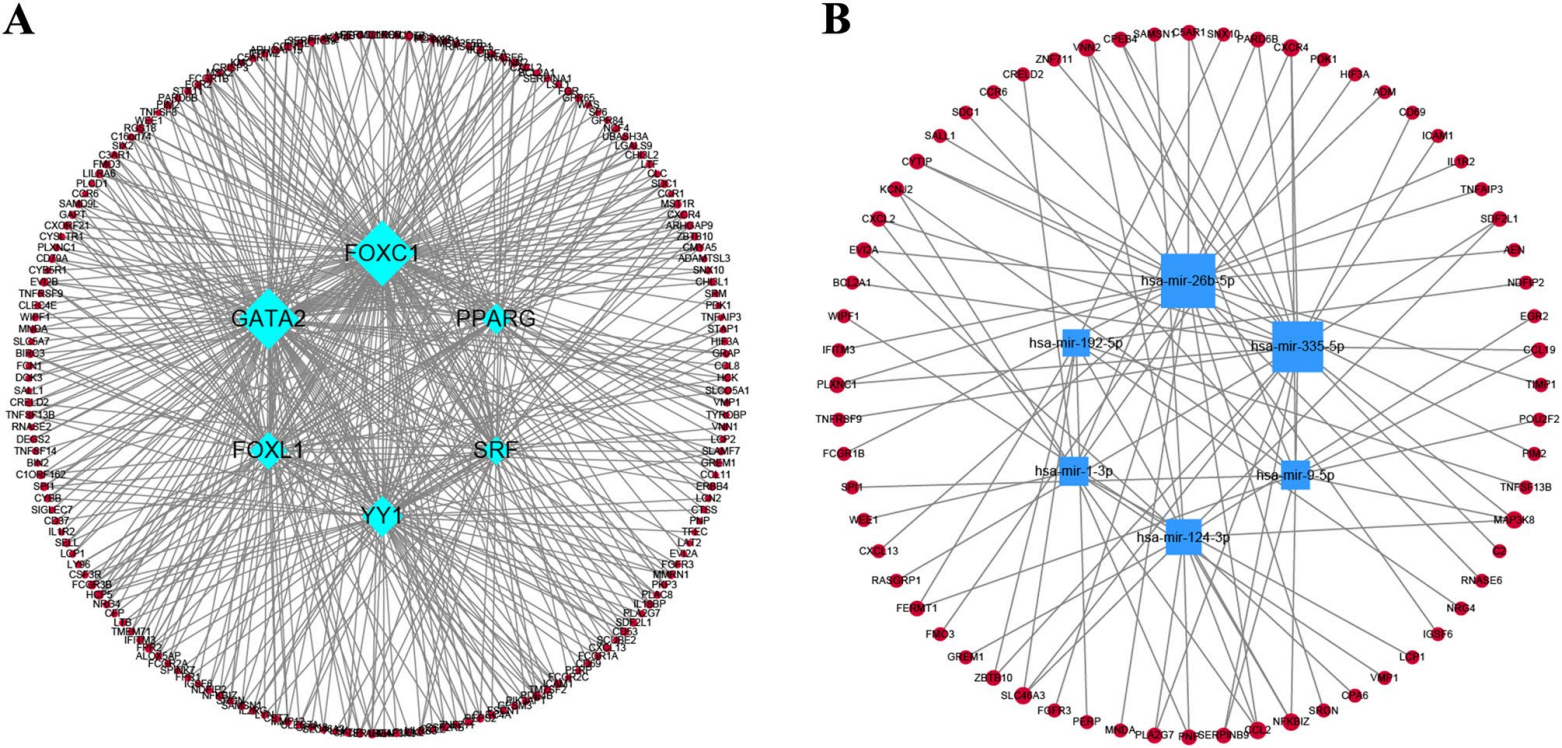
The network of DEG-TF and DEG-miRNA regulatory interactions. (**A**) TFs are represented here as diamond nodes, while gene symbols operate as circle nodes to interact with TFs. (**B**) The square node in this case denotes the circle-shaped interaction between gene symbols and miRNAs.

The interaction array of TFs and common DEGs is depicted in Fig. 6A. Node degrees, indicating interconnections, designate influential network hubs. Blue diamonds depict TFs, red circles signify DEGs, and node size mirrors the degree. According to their degrees, FOXC1, GATA2, YY1, FOXL1, PPARG, and SRF exhibited the most heightened involvement in the TF network, accentuating their prominence (Supplementary Table S2).

Figure 6B reveals miRNA regulatory interactions with shared DEGs, featuring blue squares (miRNAs) and red circles (DEGs). Notably, hsa-mir-26b-5p, hsa-mir-335-5p, hsa-mir-124-3p, hsa-mir-1-3p, hsa-mir-192-5p, and hsa-mir-9-5p had the most degrees, which emerged as the pivotal miRNAs. Details of the common miRNA regulatory networks obtained can be found in Supplementary Table S3.

### Identification of disease associations

**Figure 7:**
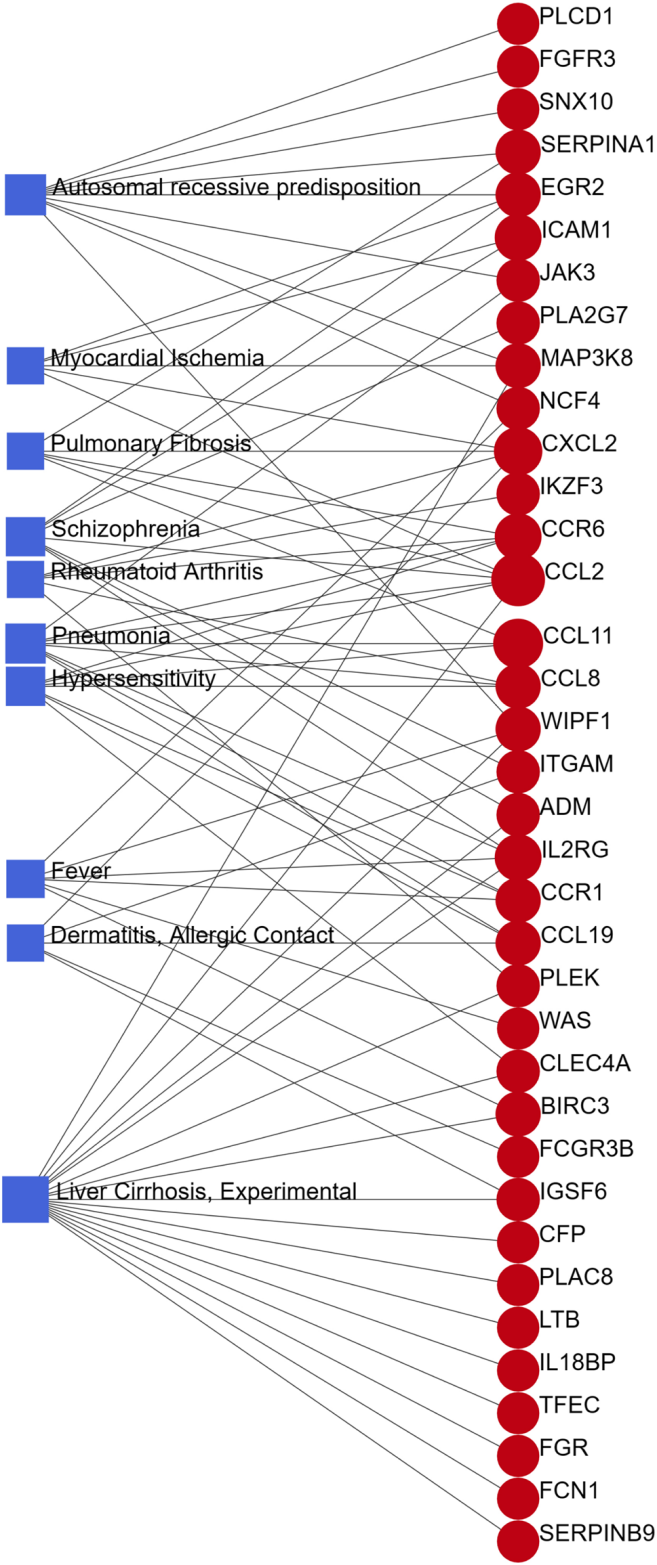
The gene-disease association network is a representation of the diseases associated with common DEGs. The circle node and its subsequent gene symbols are linked to the square node, which specifies the top 10 related diseases.

Various diseases can exhibit interrelationships by establishing connections through shared genes^37^. Gene-disease associations conducted on the Networkanalyst platform revealed intriguing links, and the Networkanalyst platform provided visualization of the results. We noticed that liver cirrhosis experimental, autosomal recessive predisposition, pneumonia, hypersensitivity, schizophrenia, fever, dermatitis allergic contact, rheumatoid arthritis, pulmonary fibrosis, and myocardial ischemia emerged as entwined with our common genes (Fig. 7). Notably, most of these diseases are closely related to inflammation or immune responses in the body.

### Exploration of potential drugs

Employing the DSigDB module within the EnrichR database, candidate drugs were discerned via common DEGs. The most pertinent drugs emerged through the evaluation of p-values that bestow promise for potential therapeutic avenues targeting COVID-19 and IC and invite further exploration. The ten most pertinent drugs were denoted as Vorinostat, Mebendazole, Trichostatin, Nickel Sulfate, Tretinoin, Demecolcine, Emetine, Isotretinoin, Methotrexate, and Phorbol 12-myristate 13-acetate (Table 2).

**Table 2:** Gene–drug interaction enrichment analysis identifies the leading ten drug candidates.

Term	P-value	Chemical Formula	Structure
Vorinostat	1.48E−15	C14H20N_2_O_3_	
Mebendazole	7.59E−14	CHN_3_O_3_	
Trichostatin	1.34E−13	C17H22N_2_O_3_	
Nickel sulfate	2.05E−11	NiSO_4_	
Tretinoin	3.42E−11	C20H28O_2_	
Demecolcine	3.46E−11	C21H25NO_5_	
Emetine	7.06E−11	C29H40N_2_O_4_	
Isotretinoin	8.08E−11	C20H28O_2_	
Methotrexate	1.04E−10	C20H22N8O_5_	
Phorbol 12-myristate 13-acetate	5.23E−10	C36H56O_8_	

### Immunocyte infiltration analysis and its correlation with hub genes

**Figure 8:**
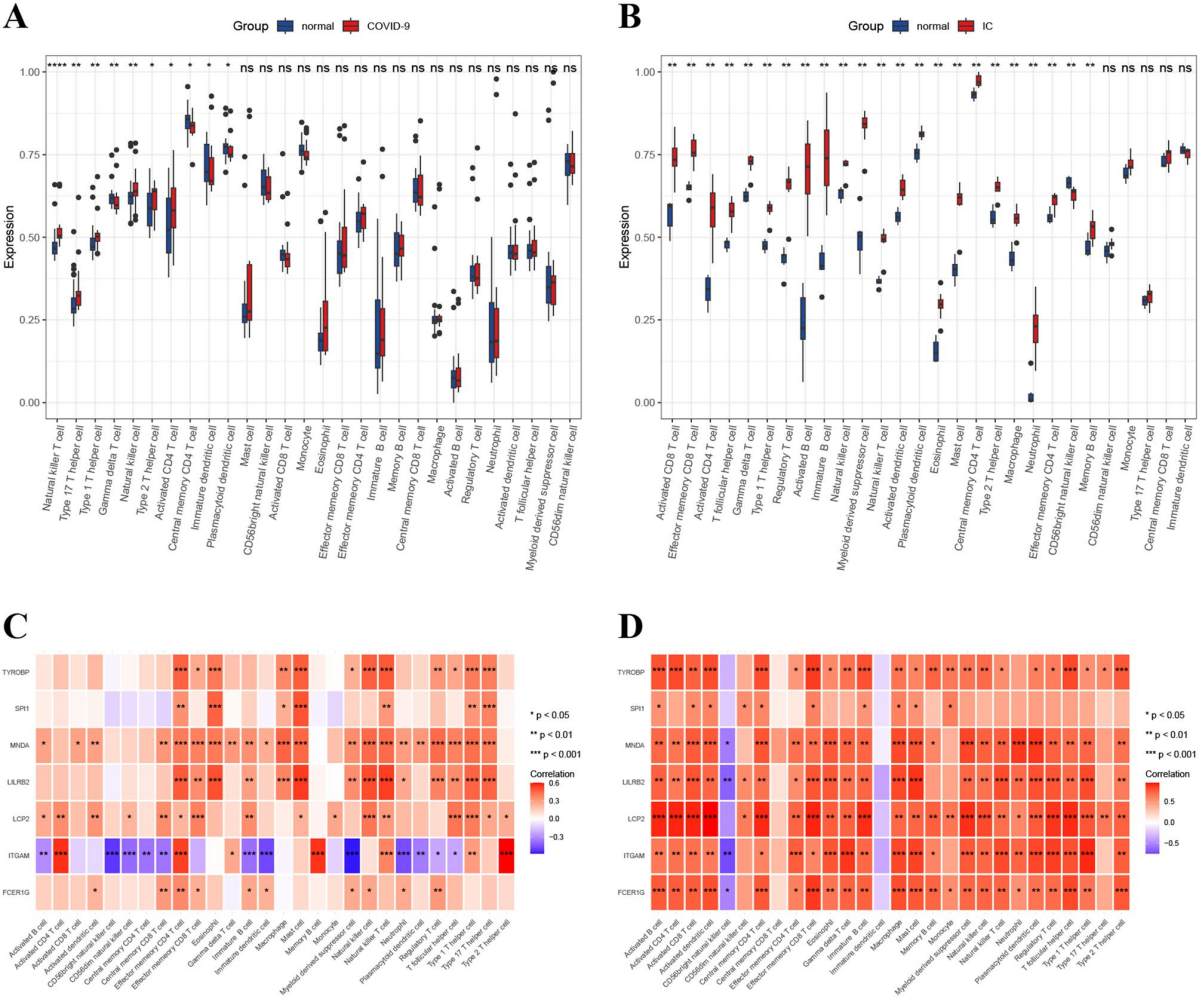
Immune infiltrations were related to the hub genes. (**A**, **B**) Immune cell infiltration analysis of datasets GSE147507 and GSE11783. (**C**, **D**) Correlation analysis of immune-related hub genes and immune cell infiltration. *p < 0.05, **p < 0.01, and ***p < 0.001.

Based on the foregoing functional enrichment analysis, it was indicated that immune response and inflammation were crucial factors in the development of IC and COVID-19. SsGSEA was utilized to unveil distinct immunological environments in the two disorders, comprising 28 subcategories of immune cells. The COVID-19 dataset showed a correlation between immune cells subtypes, including activated CD4 T cells, type 1 T helper cells, type 17 T helper cells, type 2 T helper cells, natural killer cells, and natural killer T cells (Fig. 8A). The progression of IC involved various types of immune cells, such as activated CD8 T cells, effector memeory CD8 T cells, activated CD4 T cells, central memory CD4 T cells, effector memeory CD4 T cells, T follicular helper cells, gamma delta T cells, type 1 T helper cells, type 2 T helper cells, regulatory T cells, activated B cells, immature B cells, memory B cells, natural killer cells, myeloid derived suppressor cells, natural killer T cells, activated dendritic cells, plasmacytoid dendritic cells, macrophage, eosinophil, mast cells, and neutrophil (Fig. 8B). Furthermore, we examined the relationship between the infiltration levels of 28 immune cells and the hub genes in individuals with IC and COVID-19. The strong relationship between these 7 genes and the infiltration levels of numerous immune cells in both datasets is verified by Fig. 8C and D, indicating their significant function in immune regulation. These findings demonstrate alterations in the typical immune response of individuals with IC and COVID-19, and suggest that seven specific genes may play a role in regulating the immunological milieu in these complicated circumstances.

### Diagnostic performance and correlation analysis of hub genes

**Figure 9:**
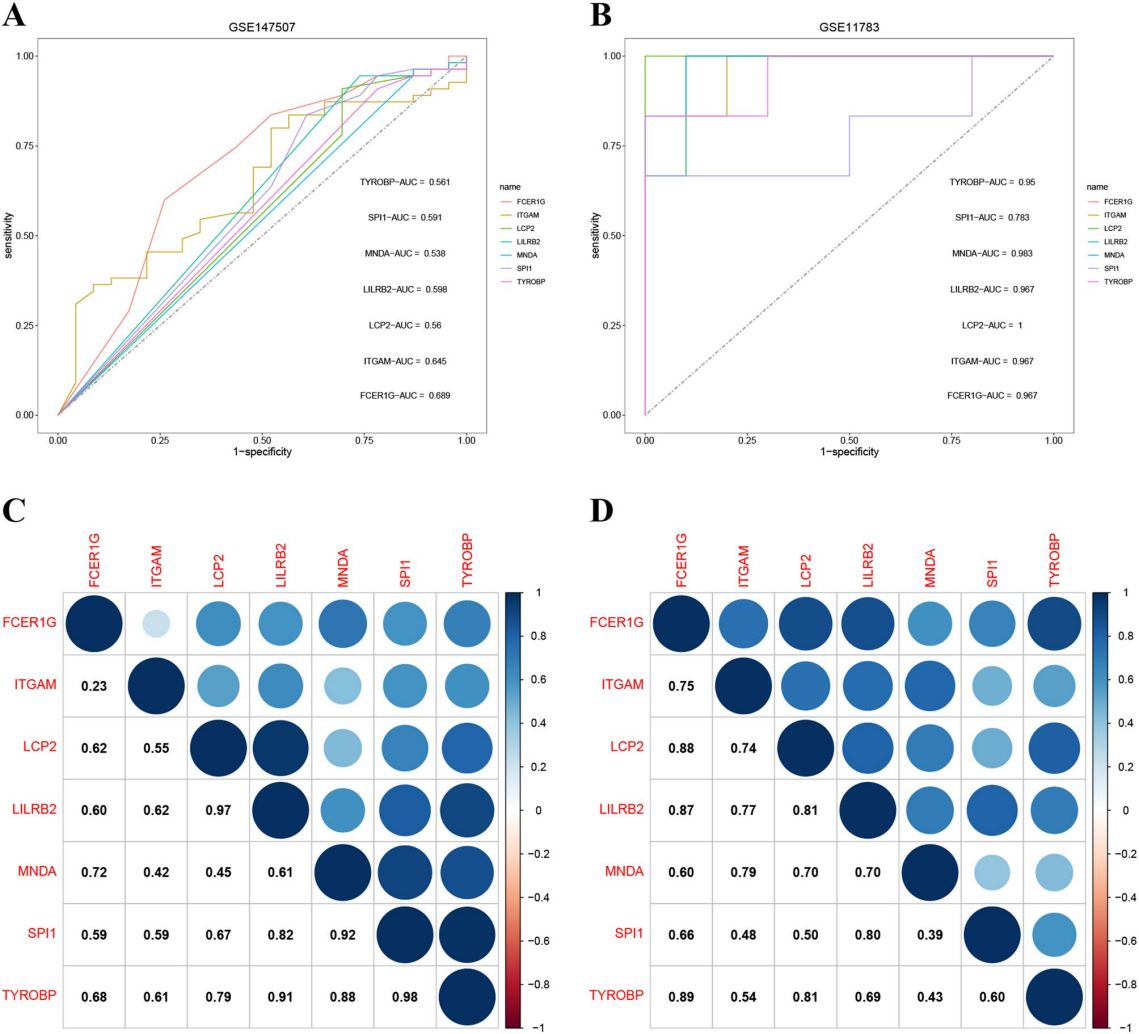
The validation of the diagnostic efficacy of seven immune-related hub genes, as well as their expression correlation. (**A**, **B**) ROC curves of seven immune-related hub genes in the datasets GSE147507 and GSE11783. (**C**, **D**) Correlations between the seven hub genes that are reciprocal.

We then gauged the diagnostic efficiency of the hub genes using ROC curves and expression data. In the COVID-19-related dataset GSE147507, TYROBP, SPI1, MNDA, LILRB2, LCP2, ITGAM, and FCER1G exhibited AUCs of 0.561, 0.591, 0.538, 0.598, 0.56, 0.645, and 0.689, respectively (Fig. 9A). Moreover, in the IC-related dataset GSE11783, these seven pivotal genes all exceeded 0.7 in AUC value (Fig. 9B). Besides, strong positive correlations emerged among their expression levels, such as SPI1/TYROBP (r = 0.98), LCP2/LILRB2 (r = 0.97), and TYROBP/TYROBP (r = 0.92) in GSE147507 (Fig. 9C), and FCER1G/TYROBP (r = 0.89), FCER1G/LCP2 (r = 0.88), and FCER1G/LILRB2 (r = 0.87) in GSE11783 (Fig. 9D). Overall, these findings indicate that the seven hub genes have a strong capacity to identify IC, which suggests a viable approach for managing those patients with CAC.

**Figure 10:**
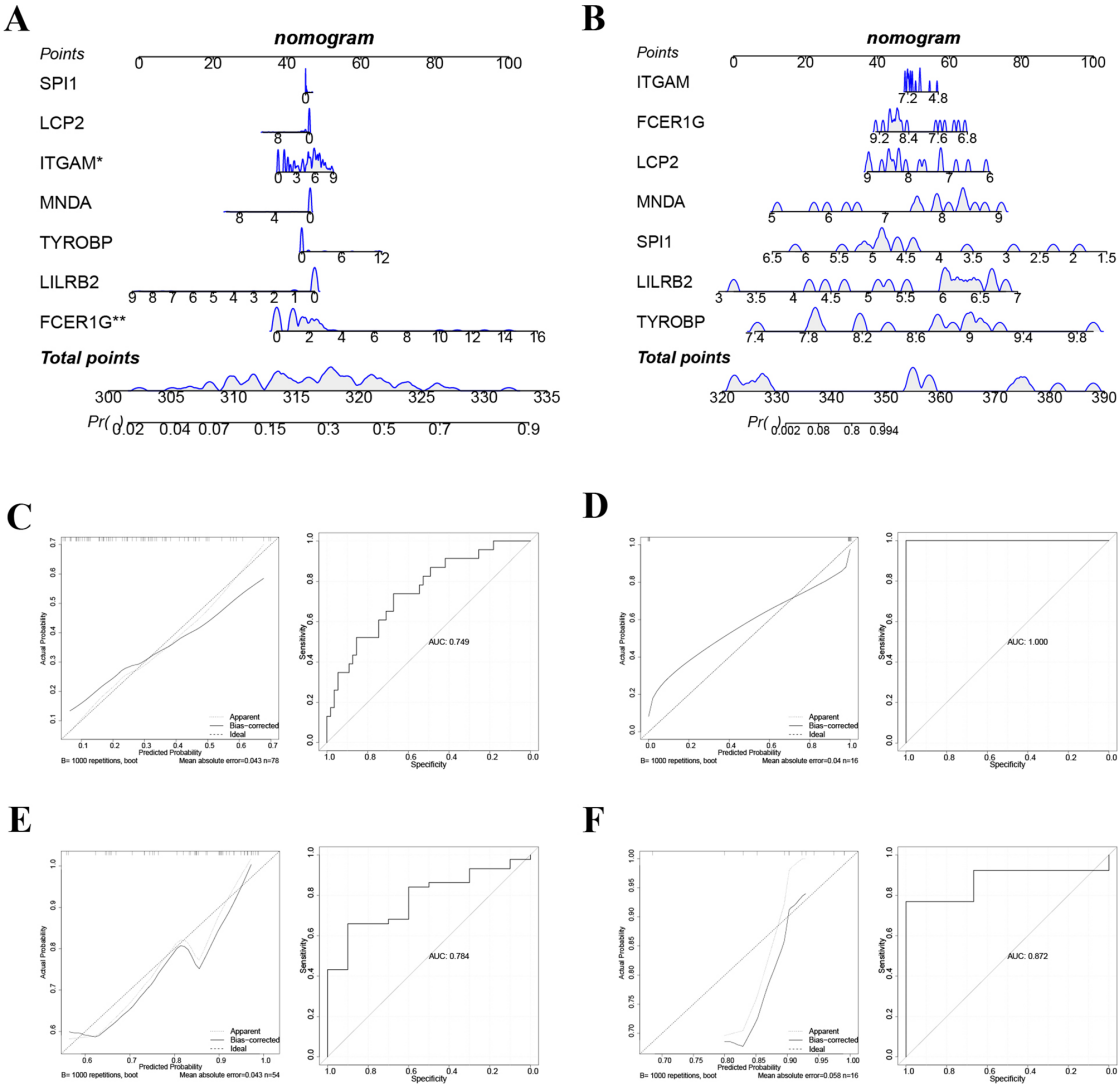
Construction and validation of COVID-19 and IC diagnostic column line graph models. (**A**) Column line graphs are used to predict the occurrence of COVID-19. (**B**) Column line graphs are used to predict the occurrence of IC. (**C**) Calibration and ROC curves to assess the diagnostic value of the COVID-19-related column line graph model in the GSE147507 dataset. (**D**) Calibration and ROC curves to assess the diagnostic value of the IC-related column line graph model in the GSE11783 dataset. (**E**) GSE171110 dataset to verify the calibration and ROC curves for COVID-19. (**E**) GSE57560 dataset to verify the calibration and ROC curves for IC.

In addition, we utilized the RMS package to construct Nomogram models for COVID-19 and IC based on the seven signature genes (Fig. 10A,B). We assessed the predictive performance of these models using calibration and ROC curves. The calibration curves in the training sets demonstrate minimal disparities between the actual and projected illness risks for both diseases, suggesting a high level of accuracy in both column line graph models. The ROC curve analysis demonstrates that the diagnostic model for COVID-19 has an AUC value of 0.749 in the training set, whereas the diagnostic model for IC has a perfect AUC value of 1 in the training set (Fig. 10C,D). These results indicated the strong predictive capabilities of both diagnostic models. Furthermore, the predictive value of these models surpasses that of each of the seven hub genes. The validation conducted on the validation sets GSE171110 and GSE57560 further substantiated the above findings (Fig. 10E,F).

## Discussion

Since the emergence of the COVID-19 pandemic in 2019, global confirmed cases have exceeded 700 million, leading to intricate complications that strain patient diagnosis and treatment^38^. The typical clinical symptoms of this disease include fever, dry cough, and fatigue, along with chills, headaches, sore throats, shortness of breath, and difficulty breathing^39^. Remarkably, some patients manifest additional symptoms, including urgency, frequency, nocturia, and hematuria, contributing to elevated mortality rates of up to 25%^40^. Researches have demonstrated that SARS-CoV-2 infection increases the production of several pro-inflammatory cytokines, and severe cases of COVID-19 often exhibit disruptions in the immune system^41,42^. Moreover, the clustering of immune cells has a significant impact on the development of cystitis^43^. In addition, the increased ACE2 expression in the bladder makes it more susceptible to SARS-CoV-2 infection, emphasizing the organ's susceptibility during COVID-19^12,44^. The present study focuses on investigating the relationship between cystitis and COVID-19 by utilizing bioinformatics and machine learning methods to identify probable molecular processes.

Firstly, we delineated the DEGs associated with IC and COVID-19. A total of 198 common genes were found to be associated with the development of both diseases. Further functional enrichment analysis highlighted their pronounced correlation with immunological and inflammatory pathways. A recent study has discovered that the coordinated activation of immune cells and the modulation of inflammatory responses are crucial in the development of COVID-19^45^. SARS-CoV-2 has the ability to directly stimulate immune cells, such as mast cells, which leads to the release of inflammatory substances. This process contributes to the occurrence of cytokine storms, which are associated with increased mortality, multi-organ failure, acute respiratory distress syndrome, and intravascular coagulation in severe cases of COVID-19^46,47^. Moreover, once mast cells become activated, they can exert their effects on the nearby smooth muscle and vascular epithelium in the bladder by releasing histamine, interleukin-6, and tumor necrosis factor-α. Furthermore, the elevation of cytokines and chemokines might worsen the stimulation and enlistment of mast cells, intensifying the advancement of localized inflammation and impeding the effectiveness of therapy^48,49^. These findings suggest that the regulation of the immune response and the secretion of cytokines may serve as the connection or association between cystitis and COVID-19.

We constructed PPI networks based on common DEGs, revealing important functional proteins and potential biomarkers between COVID-19 and cyclitis. Furthermore, utilizing the four methods of the CytoHubba plugin, we identified a total of seven hub genes. LILRB2, in combination with ANGPTL8, can enhance the migration and inflammatory activation of monocyte-derived macrophages^50^. After being cleaved by caspases, MNDA builds up in the cytoplasm, aiding in the breakdown of the antiapoptotic protein Mcl-1 and encouraging neutrophil apoptosis^51^. SPI1 serves as a transient regulator of early T cell precursors, altering the activity of pre-T cell genes during their development^52^. TYROBP activates the PI3K/AKT pathway, leading to the upregulation of inflammatory markers^53^. Induced by stst1, Lcp2 can further activate the transcription factors NFAT and NF-κB^54,55^. FCER1G codes for the Fc receptor γ chain, which is present in various types of immune cells, aiding in the removal of pathogens and antigens and also promoting abnormal immune responses such as IgE-dependent allergies by interacting with crystalline particles of immunoglobulins^56–58^. ITGAM enhances leukocyte identification of endothelial ICAM, allowing for attachment and subsequent migration of leukocytes from endothelial cells to the subendothelial area^59^. Studies have demonstrated that ITGAM is essential for the development of inflammation during pulmonary infection and is associated with enduring pulmonary complications in individuals with COVID-19^60,61^. Furthermore, in IC, the expression of CD11b, which is encoded by ITGAM, is reduced. It can be mitigated by TAK-242, a specific antagonist of TLR4^62^. These findings provide additional support for our identified hub genes, indicating their involvement in the development of IC and COVID-19, as well as their potential as targets for therapy.

Next, we employed NetworkAnalyst to investigate the transcriptional regulation of common DEGs between COVID-19 and cystitis, which focused on examining the relationships between TF, miRNA, and genes. It is reported that Foxc1, YY1, gata2, and FoxL1 play an important role in COVID-19^63,64^. PPARG has potential anti-inflammatory effects in a wide range of inflammation-related diseases. Jaclyn Estes et al.^65^ validated the capacity of PPARG to ameliorate bladder symptoms under the stimulation of pioglitazone in IC. Moreover, Gianandrea Pasquinelli et al.^66^ concluded the crucial function of PPARG in suppressing the cytokine storm by reducing the activity of pro-inflammatory cytokines in COVID-19. Recent researches conducted by Alireza et al.^67^ and Zofia et al.^68^ suggested that the robust hybridization of hsa-mir-26b-5p and hsa-mir-124-3p with ACE2 is involved in controlling the identification and assault of SARS-CoV-2. Hsa-mirna-335-5p upregulates autophagy-related factors to alleviate inflammation^69^. Furthermore, the suppression of hsa-mir-1-3p's activity towards phosphoribosylaminoimidazole succinocarboxamide synthetase (paics) hinders the mitotic process involving mechanical function in non-small cell lung cancer (NSCLC)^70^. Additionally, hsa-mir-192-5p and hsa-mir-9-5p regulate the proliferation of tumor cells and their transformation into fibrotic tissue^71,72^. Our findings suggest that these miRNAs and TFs may play a role in the immune response and inflammatory processes associated with COVID-19 and IC. However, further elucidation of their functions in the aforementioned processes is needed.

We also established a gene-disease relationship network to elucidate the correlation between the DEGs and different diseases. The findings suggested that these genes are mostly associated with inflammatory disorders and respiratory conditions, such as pneumonia, hypersensitivity, dermatitis, allergic contact, rheumatoid arthritis, and mycocardial ischemia. Pneumonia emerges as a quintessential complication of COVID-19, with severe cases often accompanied by varying degrees of fibrosis^73^. Second, Sars-cov-2 exhibits a notable affinity for the liver and the biliary system^74^, directly causing mitochondrial swelling and stem cell apoptosis, which leads to liver impairment^75^. The mortality rate among COVID-19 patients with coexisting cirrhosis also exhibits a significant escalation^76^. Moreover, during the ongoing COVID-19 pandemic, there have been indications of emerging mental disorders following exposure to the virus^77^. Individuals suffering from severe mental disorders, such as schizophrenia, typically face increased susceptibility to infection and more serious consequences^78^.

Previously, researchers discovered VV116 and paxlovid as possible treatment agents for COVID-19. These drugs have shown prolonged clinical recovery and significant decreases in mortality risk among patients with COVID-19^79^. Nevertheless, there is currently no empirical evidence supporting the efficacy of any medication in treating COVID-19 or preventing SARS-CoV-2 infection in patients with cystitis. Employing the EnrichR database, we sieved through a pool of 10 promising drugs. In a previous computational study, Debajit Dey et al.^80^ discovered that retinoic acid has the ability to reduce the activity of the sars-cov-2 E virus channel protein, hence affecting viral assembly. Mahmoud Ahmed^81^ discovered Mebendazole's inhibitory ability against the main protease (MPRO) of sars-cov-2 using molecular modeling. According to a retrospective study, mebendazole-treated patients exhibited markedly abbreviated hospital stays^82^. Besides, Vorinostat, a potent histone deacetylase (HDAC) inhibitor, has shown efficacy in treating lymphoma and human papillomavirus (HPV) infections^83^. HDAC inhibitors reduce neurotoxicity by suppressing pro-inflammatory cytokines, including IL-6 and TNF. According to a previous studies, it has the ability to defend against neural impairment in cases when infection with coronavirus type 2 results in the emergence of severe acute respiratory syndrome^84^. Furthermore, Subhash et al.^85^ revealed the effectiveness of HDAC inhibitors in restoring DNA damage repair, reprogramming detrusor function, and preventing hemorrhagic cystitis. Consequently, considering the preventive use of HDAC inhibitors, like Vorinostat, in severely affected COVID-19 patients patients is expected to halt the progression of CAC improve survival chances.

Given the observation that immune disorders may represent a prevalent pathological mechanism and molecular alteration in both IC and COVID-19, we employed the ssGSEA algorithm to accurately assess the correlations of the levels of infiltration of 28 immune cells with the two illnesses as well as with the seven crucial genes in the two diseases. Our findings demonstrate a notable increase in the infiltration of T cells, mast cells, and NK cells in both disorders, highlighting their pivotal function in modulating the immunological milieu. T cells, central to adaptive immunity, orchestrate cellular immune responses^86^. The activated CD8 T cells orchestrate pro-inflammatory cytokine secretion and infected cell death through perforin^87^, while CD4 T cells indirectly guide infection clearance by modulating CD8 T cells, neutrophils, and B cells activity^88^. Furthermore, CD4, CD8, and γδ T cells infiltrating bladder tissues could potentially underpin bladder tissue injury. Alba Grifoni et al. discovered that CD8 T cells possess the capacity to identify the spike protein of SARS-CoV-2^89^. The bladder tissue harbors the specific site of SARS-CoV-2 infiltration, ACE2, indicating the potential for T cells to mediate bladder injury in patients during COVID-19 infection. In addition, after being activated by TNFα via TNFR1, mast cells release a substantial amount of TNFα, which contributes to the persistent inflammation^90,91^, that drives cytokine storms in COVID-19^46^, and worsens the local inflammation associated with cystitis^49^. Moreover, NK cells, as lymphocyte subtypes, have the ability to initiate spontaneous cytotoxicity, annihilating virus-infected cells via CD16-mediated antibody-dependent cytotoxicity (ADCC)^92^. A study suggested that early acute SARS-CoV-2 infection coincides with heightened chemokine levels (such as CCL3, CCL3L1, etc.), thereby attracting NK cells from the circulation to infected areas^93^. Robert et al. confirmed the presence of increased NK cells in IC through immunohistochemistry^94^, which is consistent with our findings. We hypothesized that the migration of immune cells may lead to or aggravate the occurrence and development of cystitis during the infection with COVID-19.

Previous research has explored the genetic factors associated with COVID-19 or cystitis. Nevertheless, the complex relationship between these two disorders has not been fully elucidated. To bridge this gap, we untangled their shared molecular underpinnings via bioinformatics. However, our study has certain limitations. All of the data we used sourced from public databases, for which we cannot evaluate input errors. Besides, our findings—encompassing shared DEG identification, regulatory network delineation, and candidate drug identification—emanate from bioinformatics analyses. As such, the precise roles and mechanistic nuances of hub genes within immune and inflammatory processes warrant further experimental or clinical validation.

## Conclusion

By employing bioinformatics analysis, we investigated the connectivity between COVID-19 and cystitis based on the shared DEGs. Functional evaluations identified immune responses and cytokines as common pathways. We established the regulatory network connecting the common DEGs with TFs and miRNAs. Drugs that have been identified through protein-drug interactions show promise as possible therapies for CAC. Genes produced from the PPI network offer new and innovative targets for therapy. Subsequently, by utilising CytoHubba, a total of 7 crucial genes were identified and two Nomogram models were constructed to forecast the probabilities of contracting COVID-19 and IC. The models had strong performances as their AUCs exceeded 0.7 in both the training and validation sets. In summary, our work provides theoretical principles and innovative perspectives on the CAC inquiry.

### Supplementary Information

Supplementary Figure S1.

Supplementary Table S1.

Supplementary Table S2.

Supplementary Table S3.

## Data Availability

The data analyzed in this study were obtained from publicly available databases. The GSE147507, GSE11783, GSE171110 and GSE57560 chips from the GEO database (https://www.ncbi.nlm.nih.gov/gds).
