# Supplementary material for: Identification of shared pathogenetic mechanisms between COVID-19 and IC through bioinformatics and system biology
Source: Sci Rep. 2024 Jan 24;14:2114. doi: 10.1038/s41598-024-52625-z (PMC10808107; doi:10.1038/s41598-024-52625-z)
Supplement: Supplementary file 1 — Supplementary Figure S1. [file 41598_2024_52625_MOESM1_ESM.docx]

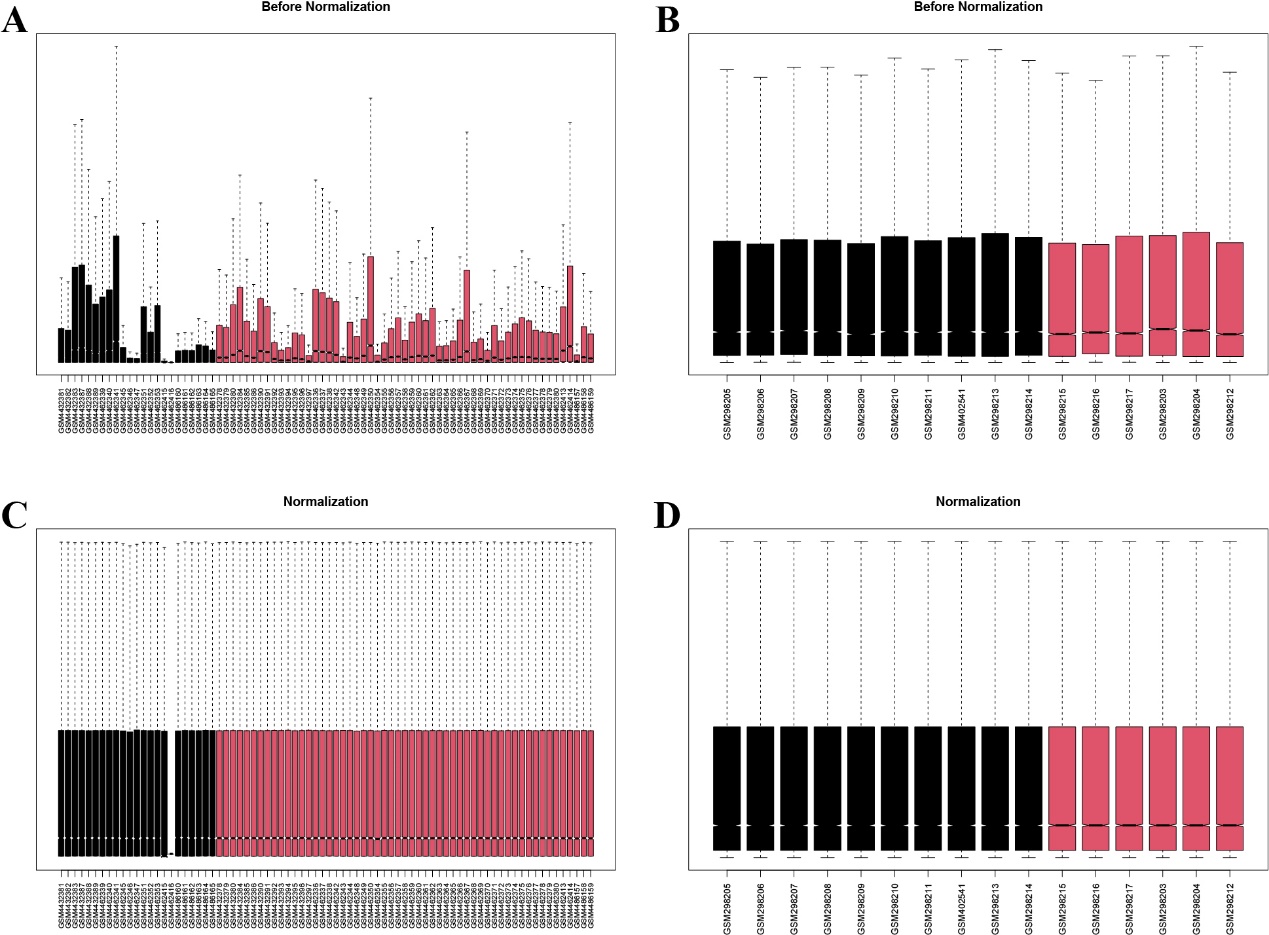


Supplement Fig S1.

The gene expression schematic of the chosen GEO datasets before to and following standardisation. (A) The GSE147507 dataset was not standardised. (B) The GSE11783 dataset was not standardised. (C) The GSE147507 dataset has undergone standardisation. (D) The GSE11783 dataset has undergone standardisation.
